# Change of Positive Selection Pressure on HIV-1 Envelope Gene Inferred by Early and Recent Samples

**DOI:** 10.1371/journal.pone.0018630

**Published:** 2011-04-19

**Authors:** Izumi Yoshida, Wataru Sugiura, Junko Shibata, Fengrong Ren, Ziheng Yang, Hiroshi Tanaka

**Affiliations:** 1 School of Biomedical Sciences, Tokyo Medical and Dental University, Tokyo, Japan; 2 AIDS Research Center, National Institute of Infectious Diseases, Tokyo, Japan; 3 Clinical Research Center, National Nagoya Medical Center, Nagoya, Japan; 4 Department of Biology, University College London, London, United Kingdom; University of Wyoming, United States of America

## Abstract

HIV-1 infection has been on the rise in Japan recently, and the main transmission route has changed from blood transmission in the 1980s to homo- and/or hetero-sexual transmission in the 2000s. The lack of early viral samples with clinical information made it difficult to investigate the possible virological changes over time. In this study, we sequenced 142 full-length *env* genes collected from 16 Japanese subjects infected with HIV-1 in the 1980s and in the 2000s. We examined the diversity change in sequences and potential adaptive evolution of the virus to the host population. We used a codon-based likelihood method under the branch-site and clade models to detect positive selection operating on the virus. The clade model was extended to account for different positive selection pressures in different viral populations. The result showed that the selection pressure was weaker in the 2000s than in the 1980s, indicating that it might have become easier for the HIV to infect a new host and to develop into AIDS now than 20 years ago and that the HIV may be becoming more virulent in the Japanese population. The study provides useful information on the surveillance of HIV infection and highlights the utility of the extended clade models in analysis of virus populations which may be under different selection pressures.

## Introduction

Whether human immunodeficiency virus type 1 (HIV-1) has reached peak virulence or has started evolving toward attenuation is controversial, with different studies suggesting that HIV-1 virulence has been increasing [1], [2], [3], [4], [5], [6], stable [7], [8] or decreasing [9], [10]. Arien et al. [11] proposed a model in which the viral virulence can be either attenuated or increasing depending on the genetic diversity of the host population. In a human population with mixed HLA (Human Leukocyte Antigen) alleles and diverse host polymorphisms, the CTL (cytotoxic T lymphocyte) response of the recipient may recognize a different set of HIV-1 epitopes from the donor, so that new mutations in viral epitopes may be necessary for CTL escape, which may cause a reduced viral fitness and lead to HIV-1 attenuation. In contrast, in a homogenous human population with little HLA and genetic diversity, the virus with acquired escape mutations from the donor may escape the CTL response of the recipient as well, so that the virus may become even more virulent leading to rapid disease progression.

In Japan, the number of HIV-1 infected individuals is increasing in recent years, and the main route of infection has changed from blood transmission in the 1980s to homo- and/or heterosexual transmission in the 2000s. The change of transmission routes may affect the pathogenicity of the virus circulating in the population. However, the lack of early viral samples makes it difficult to study possible changes in viral pathogenicity. In this study, we sequenced the full-length *env* gene from HIV-1 samples collected in the late 1980s from six Japanese subjects. In order to investigate possible changes in viral diversity and pathogenicity, we also sequenced the *env* gene from HIV-1 samples collected in the 2000s from 10 Japanese subjects.

The *env* gene is the fastest-evolving gene in the HIV-1 genome [12], [13], [14], [15]. While new mutations in the *env* gene may allow the virus to escape from host immune response, they may also disrupt the function of *env* as the viral envelope. Such conflicting selective pressures shape the evolutionary dynamics of the virus and its population diversity. In this study, we are interested in the extent by which the *env* gene has been able to diversify at the population and individual levels, and whether the virus has been undergoing adaptive evolution. Since CD4 counts and information on viral load were unavailable for the early samples, we employed computational approaches to infer diversity changes and to detect positive selection acting on the *env* gene. In particular, we are interested in whether positive selection pressure differs between the early and recent samples and between within-host and between-host evolution [16], [17]. We are also interested in detecting sites in the *env* gene targeted by the human immune system.

## Results

### Sequences from seven early subjects and 10 recent subjects were successfully obtained

Information concerning the transmission routes and sampling times of all subjects are shown in Table 1. Since not all the early samples were preserved in ideal conditions, we sequenced only seven of the 16 early subjects. By using Subtype Reference Alignments [18], the viral samples from one of seven early subjects were identified as subtype C, while all other early subjects as well as all recent subjects were confirmed to be subtype B. We thus removed the subtype C samples from our analysis. Forty-four sequences for the early group were obtained from PBMC (peripheral blood mononuclear cell) samples as the blood plasma samples could not be amplified by PCR, while 98 sequences for the recent group were obtained from blood plasma samples. Only 2 sequences were collected from each of subjects 3 and 31 in the early group. In total, 142 full-length *env* gene sequences were obtained from the 16 subjects and used for further analysis. These sequences have been deposited in the DDBJ/EMBL/GenBank databases under accession numbers AB588196-AB588337 (142 entries).

**Table 1:** Clinical information for the 16 subjects.

Subject No.	Transmission route	Infection time	Sampling date
From 1980s			
02	Blood products	Unknown	1988
03	Blood products	Unknown	1988
31	Homosexual	Unknown	1989
33	Heterosexual	Unknown	1989
40	Blood products	Unknown	1989
60	Homosexual	Unknown	1989
From 2000s			
6657	Homosexual	2004	2005
6739	Heterosexual	2000	2005
6826	Homosexual	2000	2005
6871	Homosexual	2004	2005
6946	Heterosexual	2005	2005
7015	Heterosexual	2000	2005
7060	Homosexual	2000	2005
7259	Heterosexual	Unknown	2006
7353	Heterosexual	Unknown	2006
7374	Homosexual	2004	2006

As the codon-based analysis assumes no recombination within the sequence, we run the program RIP 3.0 to detect possible recombinants. The results showed that none of the 142 subtype B sequences was recombinant with other subtypes. For co-receptor usage, WebPSSM program predicted all samples to be CCR5-using viruses.

### No significant difference in within-host diversity between the two groups

**Figure 1:**
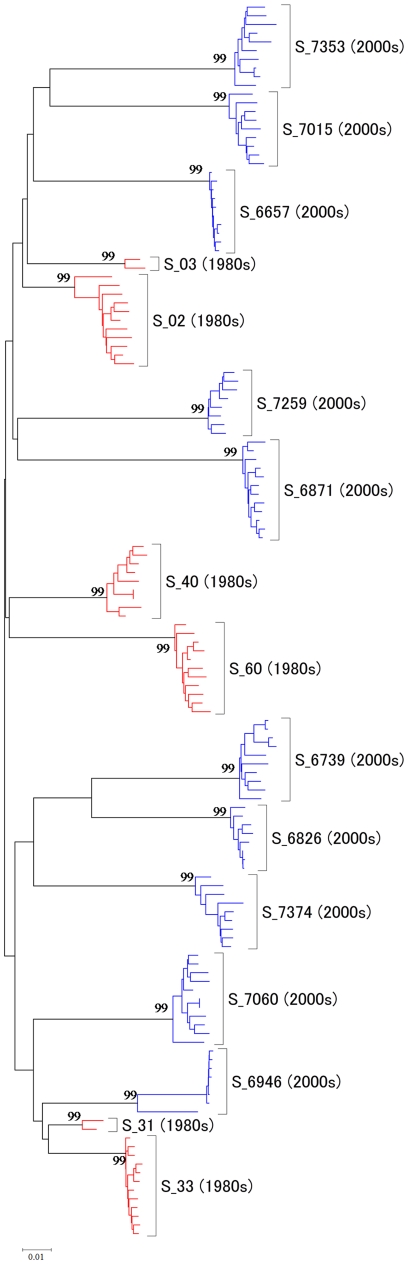
Unrooted phylogenetic tree of 142 *env* gene sequences from 16 Japanese subjects. The tree was constructed by using the neighbour-joining method (MEGA4). The 16 subjects are indicated as S_subject-number (Table 1). Viral samples from the 1980s are indicated in red while those from the 2000s are in blue. The higher bootstrap values (>70%) for major nodes were shown on the tree.

The phylogenetic tree of 142 *env* sequences was reconstructed using the Neighbor-joining method in MEGA4 [19] under the K80+G model [20] (Fig. 1). This tree is used in the codon-based maximum likelihood (ML) analysis. The robustness of our results to the tree topology is examined later, by duplicating the analysis using the ML tree under the GTR+G model (PhyML) [21]. In both the NJ and ML trees, viral sequences from the same subject formed a distinct cluster, with the between-host branches to be much longer than the within-host branches. These results are compatible with a severe bottleneck at each new infection.

The viral diversity was calculated by measuring nucleotide diversity (π) implemented in MEGA4. The between-host diversity was inferred to be 0.062 for samples of the 1980s and 0.118 for samples of the 2000s. The recent group had larger between-host diversity than the early group, which may simply reflect the accumulation of new mutations over time. The within-host diversities for the early and recent samples almost remained the same (0.012 vs. 0.011).

### Positive selection on the viral *env* gene has become weaker in the 2000s

We used the branch-site model [22], [23], [24] to detect positive selection operating on the HIV-1 *env* gene, with the selective pressure measured by the nonsynonymous/synonymous rate ratio *ω* [25]. In this model, branches on the phylogeny are partitioned *a priori* into two categories: the foreground branches which may potentially be under positive selection, and the background branches along which positive selection is assumed to be absent. On the background branches, some sites are strongly conserved with 0<*ω*_0_<1 while others are evolving neutrally with *ω*_1_ = 1. On the foreground branches some of those sites become under positive selection with *ω*_2_≥1. The parameters in the model representing the proportions of site classes and the *ω* ratios are summarized in Table 2. The branch-site test compares the null model which assumes *ω*_2_ = 1 against the alternative model with *ω*_2_≥1, with one degree of freedom used. The test allows detection of positive selection affecting the foreground branches even though most codons in the gene are under purifying selection.

**Table 2:** Parameters in the branch-site model.

Site class	Proportion	Background *ω*	Foreground *ω*
0	*p*_0_	0<*ω_0_*<1	0<*ω*_0_<1
1	*p*_1_	*ω_1_* = 1	*ω_1_* = 1
2a	(1−*p*_0_−*p*_1_)*p*_0_/(*p*_0_+*p*_1_)	0<*ω_0_*<1	*ω_2_*≥1
2b	(1−*p*_0_−*p*_1_)*p*_1_/(*p*_0_+*p*_1_)	*ω_1_* = 1	*ω_2_*≥1

Note. This is the alternative model of the branch-site test of positive selection.

The null model fixes ω_2_ = 1.

We expect the human immune system to exert selective pressure on the virus, but the pressure may differ in the 1980s and in the 2000s or between within-host and between-host evolution. We conducted four analyses in which different branches in the reconstructed tree (Fig. 1) were designated as the foreground branches: (a) 1980s-within, (b) 2000s-within, (c) 1980s-between, and (d) 2000s-between (Fig. S2). The results are summarized in Table 3. The test result was significant with *p*<1% in all four analyses, indicating that positive selection most likely operated during both within-host evolution and between-host evolution and both in the 1980s and in the 2000s. Estimates of *ω*_2_ under the model suggest that the selection pressure was stronger between hosts than within host. Interestingly, the early samples were under stronger selection even though the recent samples showed higher between-host diversity. We note that the use of *ω_2_* estimates in the branch-site model to measure the strength of positive selection may suffer from the strong correlation between estimates of *p*_2_ and *ω_2_*, as it is difficult to distinguish fewer sites under strong selection from more sites under weak selection. Thus we also calculated another heuristic measure of positive selection pressure: *p*_2_ *ω*_2_, with the expectation that both a higher *ω*_2_ and a higher *p*_2_ indicate stronger positive selection. Use of this measure instead of *ω_2_* leads to the same conclusions.

**Table 3:** Log-likelihood values and parameter estimates under the branch-site models.

Foreground branch	Δℓ	Parameter estimates	Detected sites
1980s-within	141.87	*p*_0_ = 0.616 *p*_1_ = 0.323 *p*_2_ = 0.061*ω*_0_ = 0.076 *ω*_1_ = 1 ***ω*** **_2_ = 6.598**	**3V** 151- 153A 158T **159I 161G** 163- 164- **166-** 174- **175-** 176- **178- 179- 181E 183A 184N 186T 229- 230- 234D 358N 373F** 476T **523H 527-** 530S **531T** 533V 712N **752I 798G**
2000s-within	38.05	*p*_0_ = 0.622 *p*_1_ = 0.302 *p*_2_ = 0.076*ω*_0_ = 0.076 *ω*_1_ = 1 ***ω*** **_2_ = 3.082**	12H 56K 203T **226P 228D** 231- 234D 366H **408E 415- 452N 461-** 463- 466G 476T **523H 530S 692D** 708A 811D **903L**
1980s-between	64.73	*p*_0_ = 0.633 *p*_1_ = 0.337 *p*_2_ = 0.031*ω*_0_ = 0.078 *ω*_1_ = 1 ***ω*** **_2_ = 12.5**	**144D 145L 186T** 187N 188S **204V** 404K 411G 4**20K** 447L **459- 461- 464V 470G 471S 474T** 526T 530S 708A 794P
2000s-between	58.58	*p*_0_ = 0.625 *p*_1_ = 0.337 *p*_2_ = 0.047*ω*_0_ = 0.078 *ω*_1_ = 1 ***ω*** **_2_ = 5.655**	40Q 95E 144D **145L 146R** 157S **184N** 186T **188S** 204V 341T **351Q 466G 470G 472N 473N** 530S 687L 708A **817R** 866W 907Y

Note. Positively selected sites are those with posterior probability *P*>0.95, and those with *P*>0.99 are shown in bold. Sites were numbered according to our alignment and the amino acids were from one of sequences sampled from subject 02 (02e02).

The branch-site model has the limitation that it allows only two types of branches (foreground and background) and positive selection is assumed not to occur on the background branches. For the HIV-1 *env* genes, it is possible that all branches on the tree have some sites under positive selection. The assumption of no positive selection on the background branches may affect the estimation of the strength of positive selection pressure along the foreground branches. Thus we extended the clade model C of Bielawski and Yang [26] to allow for more than two branch types (see Methods). The original model C allows for two branch types (clades) and assumes three site classes: site class 0 of conserved sites with *ω*_0_<1, site class 1 of neutral sites with *ω*_1_ = 1, and site class 2 with different selective pressures (*ω*_2_ and *ω*_3_) in the two clades. We extend this model to allow for more than two branch types, which have different rate ratios *ω*_2_ and *ω*_3_, … for site class 2 [27]. We specify five branch types in our analysis: 1980s-within, 2000s-within, 1980s-between, and 2000s-between, with all other branches grouped into one branch type. The results are summarized in Table 4. Estimates of the *ω* ratios for site class 2 for the five branch types (*ω*_2_-*ω*_6_) indicate positive selection for each branch type, but the selective pressure is weaker in the 2000s than in the 1980s. Indeed, estimates of *ω*_2_-*ω*_6_ under the clade model are in the same order as estimates of *ω*_2_ under the branch-site model when the four branch types were individually designated as the foreground branches (Table 3). The results obtained from the two analyses are thus consistent.

**Table 4:** Log-likelihood values and parameter estimates under the clade model.

	Class 0	Class 1	Class 2
Proportion	*p*_0_ = 0.591	*p*_1_ = 0.316	*p*_2_ = 0.094
All others	*ω_0_* = 0.073	*ω_1_* = 1	*ω_2_* = **5.766**
1980s-within	*ω_0_*	*ω_1_*	*ω_3_* = **4.753**
2000s-within	*ω_0_*	*ω_1_*	*ω_4_* = **2.516**
1980s-between	*ω_0_*	*ω_1_*	*ω_5_* = **7.749**
2000s-between	*ω_0_*	*ω_1_*	*ω_6_* = **4.184**

We furthermore used the extended clade model to conduct two likelihood ratio tests to examine whether the positive selection pressure has changed between the 1980s and the 2000s. The null hypothesis for the first test is that the positive selection pressure for within-host evolution is the same in the 1980s and in the 2000s (*ω*_3_ = *ω*_4_). The log likelihood was calculated either with or without this constraint, producing 2Δℓ = 32.10. The null hypothesis is thus rejected, with *p*<1%. The null hypothesis for the second test is that the positive selection pressure for between-host evolution is the same in the 1980s and in the 2000s (*ω*_5_ = *ω*_6_). This is also rejected, with 2Δℓ = 22.06, and *p*<1% and d.f. = 1.

### Positively selected sites were detected in both early and recent samples

Amino acid sites inferred to be under positive selection by the BEB (Bayes Empirical Bayes) approach under the branch-site model at the *P* = 95% level are listed in Table 3. We compared those sites with antibody binding sites in the HIV Molecular Immunology Database [28]. Some inferred sites are located in indel-rich regions and are difficult to be identified in the reference sequence HXB2. These sites were excluded. All other inferred sites were identified within at least one of epitope regions for antibody, CTL/CD8 and T-Helper/CD4 (Table 5). Furthermore, each of the four branch-site analyses detected three sites under positive selection within one of the epitopes presented by HLA alleles commonly observed in the Japanese population (with frequency >10%) [29]. Importantly, two of the three sites in the V1-V5 regions, 204V and 420K, were detected to be under positive selection along the between-1980s branches, whereas none of them was detected along the between-2000s branches.

**Table 5:** Inferred positively selected sites with epitope information in HIV immunology database.

*Foreground branch*	*Detected sites*	*Numbering in HXB2*	*Functional region*	*Epitopes*		
				CTL/CD8+	T-Helper/CD4+	Antibody
1980s-within	**358N**	**N300**	V3	+ (A2)	+	+
	**373F**	**F317**	V3	+ (A2, A*0201, A11)	+	+
	533V	I467	V5	+	+	+
	712N	L645	gp41		+	+
	**752I**	**L684**	gp41	+ (A2, A*0201, A24)	+	+
	**798G**	**D728**	gp41			+
2000s-within	56K	K46	C1	+ (A2, A11, Cw7)	+	+
	203T	T163	V2	+ (Cw8)	+	
	**226P**	**P183**	V2		+	
	366H	R308	V3	+	+	+
	**408E**	**E351**	C3		+	+
	**452N**	**N392**	V4	+	+	+
	**692D**	**N624**	gp41		+	+
	708A	S640	gp41		+	+
	811D	D741	gp41		+	+
	**903L**	**V833**	gp41	+ (A2, A*0201, A33, A*3303)	+	+
1980s-between	**204V**	**S164**	V2	+ (Cw8)	+	+
	404K	S347	C3	+ (A*0201, A11)	+	
	**420K**	**I360**	C3			
	447L	S387	V4	+ (A2)	+	+
	708A	S640	gp41		+	+
2000s-between	40Q	K33	C1	+ (A2, A*0201, B44)	+	+
	**95E**	**V85**	C1	+	+	+
	296P	P238	C2	+	+	+
	341T	T283	C2	+	+	+
	**351Q**	**E351**	C2		+	+
	687L	L619	gp41		+	+
	**708A**	**S640**	gp41		+	
	**817R**	**R747**	gp41	+ (A2)		
	**866W**	**W796**	gp41	+		+
	907Y	C837	gp41	+ (A33, A*3303)	+	+

Note. +: site reported as an epitope for antibody, CTL/CD8+ and T-Helper/CD4+ in HIV immunology database. HLA alleles observed with higher frequency (>10%) in Japanese are shown in parentheses.

### Robustness of our results

Yap et al. ([30]; see also [31]) are concerned that estimation of the *ω* ratio may be sensitive to model assumptions concerning codon usage. The authors suggested alternative ways of accommodating nucleotide/codon frequencies in models of codon substitution. Those models, like most early ones, are not based on our understanding of the biological process but are instead mathematical constructs aimed at fitting the datasets empirically. Nevertheless the potential sensitivity of our results to model assumptions is a concern. We have examined the nucleotide frequencies in our dataset, and found that they were nearly identical between the recent and early viral samples. Our analysis has been conducted under the F3×4 model of codon usage, with three nucleotide frequency parameters used for each codon position [31]. To examine the robustness of the results to assumptions concerning codon usage, we repeated the analysis under the Fcodon model (CodonFreq = 3 in codeml), which uses the 61 codon frequencies as parameters. All frequency parameters under the F3×4 and Fcodon models are estimated using the observed frequencies in the data. The results obtained under the Fcodon model are listed in Supplementary Tables S1 and S2 for the branch-site and clade models, respectively. They were very similar to those obtained in the corresponding analyses under the F3×4 model, indicating that our results may be robust to assumptions concerning nucleotide/codon frequencies.

Furthermore, we assessed the impact of the tree topology on our analysis. Instead of the NJ tree under K80+G, we also used the ML tree inferred using PhyML under GTR+G. Note that only the tree topology is used in the codon-based analysis, and branch lengths are re-estimated under the codon model. The results for the ML tree are included in Supplementary Tables S3 and S4 for the branch-site and clade models, respectively. These are highly similar to the results obtained using the NJ tree, suggesting that our conclusions may be robust to minor errors in the tree topology.

## Discussion

In this study, we contrast the changes in genetic diversity and adaptive evolution of the HIV-1 *env* gene between samples collected in the 1980s, the beginning of HIV-1 pandemics, and those collected after 2000, when the virus had spread worldwide and multiple human to human transmissions had taken place.

We sequenced HIV-1 *env* genes from seven subjects sampled in the 1980s. The samples from six of seven subjects were identified as subtype B. This finding is consistent with the observation that subtype B has been the dominating subtype in Japan since 1980s [32]. Since the early blood plasma included heparin that inhibits PCR, none of the early samples could be amplified from blood plasma and we had to use PBMC instead. We were able to obtain full-length *env* genes from both early and recent samples in this study, which gave our analysis an advantage over most previous studies on the *env* gene, which usually used only partial *env* sequences [33], [34], [35].

No significant change in within-host diversity was found after nearly two decades of evolution. The reconstructed phylogenetic tree of 142 sequences demonstrated distinctly long internal branches and short external branches, suggesting that only a small number of viruses infected the new host cell at each transmission so that these founder viruses usually are quite different among hosts. Moreover, the viruses that successfully infected new host cells are under strong selective pressure from the host immune system, which limited within-host diversification, as indicated by those small clusters on the tree. Therefore, those individual-specific mutations harbored by founder viruses may have a large impact on the within-host evolution and affect the prognosis of HIV infection.

In between-host HIV evolution, the reset of viral fitness by a genetic bottleneck may play an important role, influenced by both viral and host factors. Arien et al. [11] described two different HIV transmission scenarios for human populations with either diverse or homogeneous genetic backgrounds. We note that Arien et al. 's argument does not yet constitute a quantitative model with precise mathematical predictions. For example, it is unclear what levels of host genetic variation should cause the HIV to become attenuated or more virulent. Nevertheless, a previous study, which examined adaptive HIV-specific immune responses and viral evolution in adult monozygotic twins simultaneously infected with the same virus, provided qualitative support for the model [36]. The study found that 15 out of 17 epitopes targeted by initial CD8 T cell response were identical in each twin, indicating the concordance of adaptive immune responses in the same genetic background.

In our analysis, the between-host selective pressures were inferred to be weaker for the recent samples than for the early samples, in spite of the fact that the recent samples had higher diversity between hosts. For the early group, three out of six subjects were infected by the blood products imported from a foreign country, and the others were most likely infected with the virus from overseas nationals, representing the transmissions between the populations with different genetic backgrounds. In contrast, all the 10 subjects from the recent group represent transmission within native Japanese population. Several studies have reported that the genetic diversity in the Japanese population is very small [37], [38], [39]. In a study of gene-based SNP discovery, Haga et al. [39] found one polymorphism per 807 bp in the Japanese population, much lower than one SNP got every 272 bp for the world average [40], indicating that the Japanese population appears to be more homogeneous. Accordingly, the new CTL escape mutation upon transmission to a new host in the 2000s will be less necessary than in the 1980s. Since the CTL escape mutations in viral epitopes usually exact a cost to viral fitness [41], the HIV transmission with fewer escape mutations will have a lower cost in viral fitness. In other words, in the 2000s, the HIV may have a higher viral fitness after the transmission to a new host. The viral fitness is a key factor affecting the viral virulence which measures the capacity of a virus to cause disease [42]. Thus, we speculate that the HIV circulating in the homogeneous Japanese population may have evolved to be more virulent.

The results of Table 5 support this speculation. In spite of more sites being detected under positive selection for between-host evolution for the recent group than for the early group, none of those sites was in the V1–V5 regions, suggesting that some mutations in these epitopes, although needed for a new infection in the 1980s, may have become fixed in the population in the 2000s. In a recent study, Kawashima et al. [43] demonstrated strong evidence of HIV adaptation to HLA at a population level. The authors pointed out that the process of viral adaptation might alter currently established HLA associations with slow disease progression to AIDS. Although *pol* and *gag* genes were analyzed in their study, it appears sensible to expect similar adaptive evolution to occur on the *env* gene as well. Collection of more clinical data may shed light on this issue.

While the size of our data for the early samples is relatively small, our search in the HIV Sequence Database [44] did not locate any full-length *env* genes collected in the 1980s from the Japanese population. Instead, we found 21 partial sequences covering V2, V3, C2 and part of C3 regions of the *env* gene. These sequences were sampled from two Japanese subjects in 1988: subject 9 (AB002885-AB002894) and subject 20 (AB002922-AB002932) [45]. Subject 9 was infected through HIV contaminated blood products during 1983–1985 and subject 20 was infected through sexual transmission in 1986. Those partial sequences were aligned with the same region of our 142 full-length *env* genes using ClustalW to form a new dataset of 163 sequences, each of 693 bp. This V2–V3 dataset was analyzed in the same way as the dataset for the full-length *env* genes. The phylogeny was reconstructed using the NJ method, which was further used to detect positive selection using the branch-site and extended clade models. The reconstructed phylogenetic tree showed that the sequences obtained from each subject most tightly clustered together and were divergent from those of other subjects (Fig. S1). The codon-based analysis produced similar results to those from the full-length dataset. For example, the branch-site model suggested that the positive selection pressure along the 2000s-between branches (*ω*_6_ = 3.462) was significantly weaker than for 1980s-between (*ω*_5_ = 7.124), with 2Δℓ = 6.24. However, the V2–V3 dataset is less informative due to its smaller size (231 codons vs. 926 codons in the full-length dataset), and did not lead to significant results in some tests (results not shown).

The present study also highlights the utility of the branch-site and extended clade models in analysis of viral samples when the viral gene may be under different positive selection pressures during different time periods or in different viral populations. By using pre-specified branch types, to represent viral evolution within hosts and transmission between hosts, we can estimate *ω* ratios for different branch types and test potential differences in selective pressure during different periods of viral evolution. Notably, the extended clade model appears to be considerably useful to detect positive selection in the case that multiple viral lineages in the phylogeny may have been evolving under different selective pressures. In our study, the model performed well in both analysis of the full-length gene and the V2–V3 region datasets.

## Materials and Methods

### Viral samples

All the blood samples were preserved in the National Institute of Infectious Diseases (NIID) in Tokyo. Transmission routes and sampling times were available for all subjects but detailed clinical information was unavailable for the subjects collected in 1980s (Table 1). All subjects were Japanese and did not receive anti-HIV therapy. Both PBMC and blood plasma were available for early samples (continuously stored at −130°C), whereas blood plasma only was available for recent samples. This study was conducted according to the principles expressed in the Declaration of Helsinki. The study was approved by the Institutional Review Board of the NIID. All patients provided written informed consent for the collection of samples and subsequent analysis.

### Sequencing and phylogenetic analysis

Proviral DNA was extracted from PBMC using the QIAamp DNA Blood Mini Kit. Viral RNA was extracted from blood plasma using the MagNa Pure Compact Nucleic Acid Isolation Kit I (Roche Applied Science). RNA products were used for cDNA synthesis, using Superscript II RT Kit and random hexamer (Invitrogen).

DNA products were sequenced using the Single-Genome Sequencing (SGS) method (25). For full-length *env* gene amplification, they were PCR amplified using a set of primers (Env-6116F: 5′ - gcaatagttgtgtggwcyatag -3′ and Env-1M: 5′ - tagcccttccagtccccccttttctttta - 3′) followed by nested amplification (Env-SF1: 5′ - ctaatagaaagagcagaagacagtgg -3′ and Env-PR2: 5′ - gctsccttrtaagtcattggtct -3′). The PCR products sequenced with an ABI PRISM 3730 automated DNA sequencer (Applied Biosystems).

To identify the subtypes of the viral samples sequenced in this study, we used Subtype Reference Alignments [18]. We also tested for recombination using the program RIP 3.0 (Recombinant Identification Program) available at the same Website. Moreover, since the infection time of some subjects was unknown (Table 1), we used WebPSSM [46] to predict the co-receptor usage, as it has been known that CCR5-using viruses are important during early stages of the HIV infection, whereas CXCR4-using viruses emerge later in the progression to AIDS [47]. The default option, x4r5 matrix, was used for the prediction.

The sequences were aligned using ClustalW. First, the 142 nucleotide sequences were translated into amino acid sequences using MEGA4, which were aligned using ClustalW implemented in MEGA4. Then the aligned amino acid sequences were used to construct the nucleotide alignment. There are 926 codons (2778 bp) in the alignment. We used the software FindModel [48] to find the appropriate substitution model. Models GTR+G, HKY+G, K80+G and TrN+G were suggested to have better AIC scores and likelihoods. Then we reconstructed the phylogenetic tree using the ML method (PhyML) [21] under GTR+G and the NJ method (MEGA4) [19] under K80+G [20] (the GTR+G model is not available in MEGA4). As the resultant trees were similar, we used the NJ tree for further analysis. However, the ML tree under the GTR+G model was used to duplicate the analyses to confirm the robustness of our results to possible topological errors.

### Inferring the genetic diversity of the envelope gene

To investigate the diversity change, we inferred within- and between-host mean diversities for the 1980s and the 2000s, respectively, using the nucleotide diversity, π, implemented in MEGA4 under the K80+G model again. As the sequences are highly similar, the choice of the substitution model makes little difference to the distance calculation. For Setup Data, the viral sequences obtained from the same subject were grouped as one subpopulation. Then, the within-host diversity was calculated by Mean Diversity within Subpopulations, whereas the between-host diversity was calculated by Mean Interpopulational Diversity.

### Detecting temporal changes of positive selection pressure using codon models

We used codon models of coding sequence evolution to detect positive selection operating on the HIV-1 *env* gene. In particular, we are interested in differences in positive selection pressure on the virus between the 1980s and the 2000s, and between within-host evolution and between-host evolution. We use the branch-site model [23], [49] to detect such temporal changes in positive selection pressure, as implemented in the codeml program in the paml package [25]. The likelihood ratio test compares the branch-site model A with a null model that fixes *ω*_2_ = 1, with one degree of freedom used. This phylogeny-based analysis assumes that there is no recombination with the sequence so that all sites in the sequence are related by the same phylogenetic tree and that the substitution process has been stationary. The codon frequencies are described using the F3×4 model, which uses the nucleotide frequencies at the three codon positions to describe the codon usage, with 9 free parameters used. The frequency parameters are estimated using the frequencies observed in the data. The branch-site model makes rather stringent assumptions about the selective pressures acting on the foreground and background branches. Simulations, however, suggest that the test is quite robust if the real selective scheme is far more complex [23].

The assumption, made in the branch-site model, of no positive selection on the background branches and of only two branch types may be too restrictive for the HIV-1 *env* genes. Thus we extend the clade model C of Bielawski and Yang [26] to allow for more than two branch types. Clade model C was developed to detect divergent evolution in two clades (two branch types): with site class 0 to include conserved sites with *ω*_0_<1, class 1 to be neutral sites with *ω*_1_ = 1 and class 2 represent sites undergoing divergent evolution with different selective pressures (*ω*_2_ and *ω*_3_) in the two clades. We extend this model to allow for more than two branch types, which have different rate ratios *ω*_2_ and *ω*_3_, …, for site class 2, where 0<*ω*_2_, *ω*_3_, …<∞. The model thus accounts for conserved sites, nearly neutral sites, as well as positive-selection sites that are under different levels of selection among the branch types. We have also extended the Bayes empirical Bayes (BEB) method to calculate the posterior probabilities for the site classes when there are more than two branch types [24]. The extended clade model has been implemented in the codeml program in the paml package [25]. For the analysis of our data, we used five branch types: 1980s-within, 2000s-within, 1980s-between, and 2000s-between, with all other branches grouped into one branch type.

## Supporting Information

Figure S1 Unrooted phylogenetic tree of 163 V2–V3 region sequences from 16 Japanese subjects. The tree was constructed by using the neighbour-joining method (MEGA4). The 2 subjects found in the database are indicated as “S_88JP9” and “S_88JP20” respectively. The red represents viral samples from the 1980s group while the blue represents those from the 2000s group.
(TIF)

Figure S2 The foreground branches in the different analyses under the branch-site model. The tree topology is the same as in Fig. 1, but different foreground branches are assumed in four analysis: (a) within-1980s (red), (b) within-2000s (blue), (c) between-1980s (red), and (d) between-2000s (blue).
(TIF)

Table S1 Log-likelihood values and parameter estimates under the branch-site models using the Fcodon model (CodonFreq = 3).
(DOC)

Table S2 Log-likelihood values and parameter estimates under the clade model using the Fcodon model (CodonFreq = 3).
(DOC)

Table S3 Log-likelihood values and parameter estimates under the branch-site models using GTR+G model (CodonFreq = 2).
(DOC)

Table S4 Log-likelihood values and parameter estimates under the clade model using GTR+G model (CodonFreq = 2).
(DOC)
