# Supplementary material for: Change of Positive Selection Pressure on HIV-1 Envelope Gene Inferred by Early and Recent Samples
Source: PLoS One. 2011 Apr 19;6(4):e18630. doi: 10.1371/journal.pone.0018630 (PMC3079721; doi:10.1371/journal.pone.0018630)
Supplement: Table S1 — Log-likelihood values and parameter estimates under the branch-site models using the Fcodon model (CodonFreq = 3). (DOC) [file pone.0018630.s003.doc]

# Table S1 Log-likelihood values and parameter estimates under the branch-site models using the Fcodon model (CodonFreq = 3)

| Foreground branch | 2Δ | Parameter estimates |
| --- | --- | --- |
| 1980s-within | 299.26 | *p*_0_ = 0.607 *p*_1_ = 0.333 *p*_2_ = 0.0596 *ω*_0_ = 0.075 *ω*_1_ = 1 ***ω*_2_ = 6.82** |
| 2000s-within | 71.68 | *p*_0_ = 0.613 *p*_1_ = 0.305 *p*_2_ = 0.0545 *ω*_0_ = 0.075 *ω*_1_ = 1  ***ω*_2_ = 2.977** |
| 1980s-between | 104.76 | *p*_0_ = 0.623 *p*_1_ = 0.344 *p*_2_ = 0.0327 *ω*_0_ = 0.0781 *ω*_1_ = 1 ***ω*_2_ = 12.05** |
| 2000s-between | 10.45 | *p*_0_ = 0.617 *p*_1_ = 0.35 *p*_2_ = 0.032  *ω*_0_ = 0.0776 *ω*_1_ = 1 ***ω*_2_ = 6.27** |
