# Supplementary material for: Change of Positive Selection Pressure on HIV-1 Envelope Gene Inferred by Early and Recent Samples
Source: PLoS One. 2011 Apr 19;6(4):e18630. doi: 10.1371/journal.pone.0018630 (PMC3079721; doi:10.1371/journal.pone.0018630)
Supplement: Table S3 — Log-likelihood values and parameter estimates under the branch-site models using GTR+G model (CodonFreq = 2). (DOC) [file pone.0018630.s005.doc]

# Table S3 Log-likelihood values and parameter estimates under the branch-site models using the GTR+G tree (CodonFreq=2)

| Foreground branch | 2Δ | Parameter estimates |
| --- | --- | --- |
| 1980s-within | 296.92 | *p*_0_ = 0.869 *p*_1_ = 0.111 *p*_2_ = 0.020 *ω*_0_ = 0.0363 *ω*_1_ = 1 ***ω*_2_ = 7.625** |
| 2000s-within | 89.78 | *p*_0_ = 0.878 *p*_1_ = 0.108 *p*_2_ = 0.014 *ω*_0_ = 0.0372 *ω*_1_ = 1  ***ω*_2_ = 4.058** |
| 1980s-between | 101.68 | *p*_0_ = 0.883 *p*_1_ = 0.107 *p*_2_ = 0.010 *ω*_0_ = 0.038 *ω*_1_ = 1 ***ω*_2_ = 22.469** |
| 2000s-between | 12.18 | *p*_0_ = 0.878 *p*_1_ = 0.105 *p*_2_ = 0.017 *ω*_0_ = 0.037 *ω*_1_ = 1 ***ω*_2_ = 7.056** |
