# Supplementary material for: Change of Positive Selection Pressure on HIV-1 Envelope Gene Inferred by Early and Recent Samples
Source: PLoS One. 2011 Apr 19;6(4):e18630. doi: 10.1371/journal.pone.0018630 (PMC3079721; doi:10.1371/journal.pone.0018630)
Supplement: Table S4 — Log-likelihood values and parameter estimates under the clade model using GTR+G model (CodonFreq = 2). (DOC) [file pone.0018630.s006.doc]

# Table S4 Log-likelihood values and parameter estimates under the clade model using the GTR+G tree (CodonFreq=2)

|  | Class 0 | Class 1 | Class 2 |
| --- | --- | --- | --- |
| Proportion | *p*_0_ = 0.827 | *p*_1_ = 0.154 | *p*_2_ = 0.093 |
| All others | ω_0_ = 0.049 | *ω*_1_ = 1 | *ω*_2_ = **5.719** |
| 1980s-within | ω_0_ | *ω*_1_ | *ω*_3_ = **5.548** |
| 2000s-within | ω_0_ | *ω*_1_ | *ω*_4_ = **3.497** |
| 1980s-between | ω_0_ | *ω*_1_ | *ω*_5_ = **10.607** |
| 2000s-between | ω_0_ | *ω*_1_ | *ω*_6_ = **4.680** |

Note. The log likelihood under this model is  = –31346.08. The likelihood ratio test statistic for testing *H*_0_: *ω*_3_ = *ω*_4_ is 2Δ = 24.463 and that for testing *H*_0_: *ω*_5_ = *ω*_6_ is 2Δ = 32.93. Both are significant with *p* < 1%.
